# Interleukin 22 in Liver Injury, Inflammation and Cancer

**DOI:** 10.7150/ijbs.38925

**Published:** 2020-06-29

**Authors:** Ye Wu, Jie Min, Chang Ge, Jinping Shu, Di Tian, Yuan Yuan, Dian Zhou

**Affiliations:** 1 The First Affiliated Hospital of Anhui Medical University, Hefei, Anhui, 230022, China.; 2 The Second Hospital of Anhui Medical University, Hefei, Anhui, 230601, China.; 3 The First Affiliated Hospital of University of Science and Technology of China, Hefei, Anhui, 230022, China.

**Keywords:** IL-22, Liver injury, Inflammation, HCC, Liver regeneration.

## Abstract

Interleukin 22(IL-22), a member of the IL-10 cytokine family and is an emerging CD4+Th cytokine that plays an important role in anti-microbial defense, homeostasis and tissue repair. We are interested in IL-22 as it has the double function of suppressing or encouraging inflammation in various disease models including hepatic inflammation. As a survival factor for hepatocytes, IL-22 plays a protective role in many kinds of liver diseases, such as hepatitis, liver fibrosis, or hepatocellular carcinoma (HCC) by binding to the receptors IL-22R1 and IL-10R2. Overexpression of IL-22 reduces liver fibrosis by attenuating the activation of hepatic stellate cell (the main cell types involved in hepatic fibrosis), and down-regulating the levels of inflammatory cytokines. Administration of exogenous IL-22 increases the replication of hepatocytes by inhibiting cell apoptosis and promoting mitosis, ultimately plays a contributing role in liver regeneration. Furthermore, treatment with IL-22 activates hepatic signal transducer and activator of transcription 3 (STAT3), ameliorates hepatic oxidative stress and alcoholic fatty liver, effectively alleviate the liver damage caused by alcohol and toxicant. In conclusion, the hepatoprotective functions and liver regeneration promoting effect of IL-22 suggests the therapeutic potential of IL-22 in the treatment of human hepatic diseases.

## Introduction

Interleukin-22 (IL-22) was first identified by Dumoutier et al. in 2000 in the secretome of IL-9-stimulated thymic lymphomas 1, 2. Due to the similarity in the gene and protein structure with IL-10, IL-22 was classified as a member of the IL-10 cytokine family, which also includes other cytokines such as IL-10, IL-19, IL-20, IL-24, IL-26 and the type III interferons 3. The IL-10 family can be further subdivided into three groups based on their function; IL-10 itself, the IFN-λ subfamily and the IL-20 subfamily.

IL-22, which acts on a variety of tissues and organs such as the intestines, lungs, liver, kidney, thymus, pancreas and skin, has a lot of functions; It is worth mentioning that IL-22 acts as either a anti-inflammatory or proinflammatory cytokine in many disease models such as psoriasis, ulcerative colitis, systemic lupus erythematosus and other inflammatory diseases 3. In the liver, hepatocytes are the primary target of IL-22, and IL-22 is thought to induce hepatic production of acute-phase proteins. In liver injury, IL-22 usually induces proteins involved in protection and regeneration, such as anti-apoptotic proteins bcl-2/bcl-xl, cyclin D1, c-myc, and CDK4. Thus, IL-22 has considerable protective effects in a variety of different experimental models of liver injury, including alcohol-induced liver damage, liver ischemia-reperfusion injury. IL-22 treatment can provide critical protection against liver toxicity and liver damage caused by many different types of toxic substances 4-6. During liver inflammation, IL-22 can also provide protection to hepatocytes 7. These facts indicate that IL-22 will be a potential new target for the treatment of liver disease in the future. In this review, we summarize our current understanding of the involvement of IL-22 in liver injury, inflammation and cancer.

### IL-22

IL-22 is a cytokine largely secreted by lymphoid cells; encompass the innate and adaptive immune systems, such as group 3 innate lymphoid cells (ILC3), αβ T cells, γδ T cells and NKT cells 8. In the absence of overt infection or inflammation, ie, steady state, the main source of IL-22 is ILC3, which are present in large numbers in the mucosae of the large and the small intestine 9. Among αβ T cells, IL-22-producing human T cells are mainly Th1, Th17, and Th22 cells; approximately 33% of IL-22-producing CD4+ T cells are Th1 cells, 50% are Th22 cells, and 15% are Th17 cells 3, 7. Some reports have also found that neutrophils produce IL-22 10, 11.

**Figure 1:**
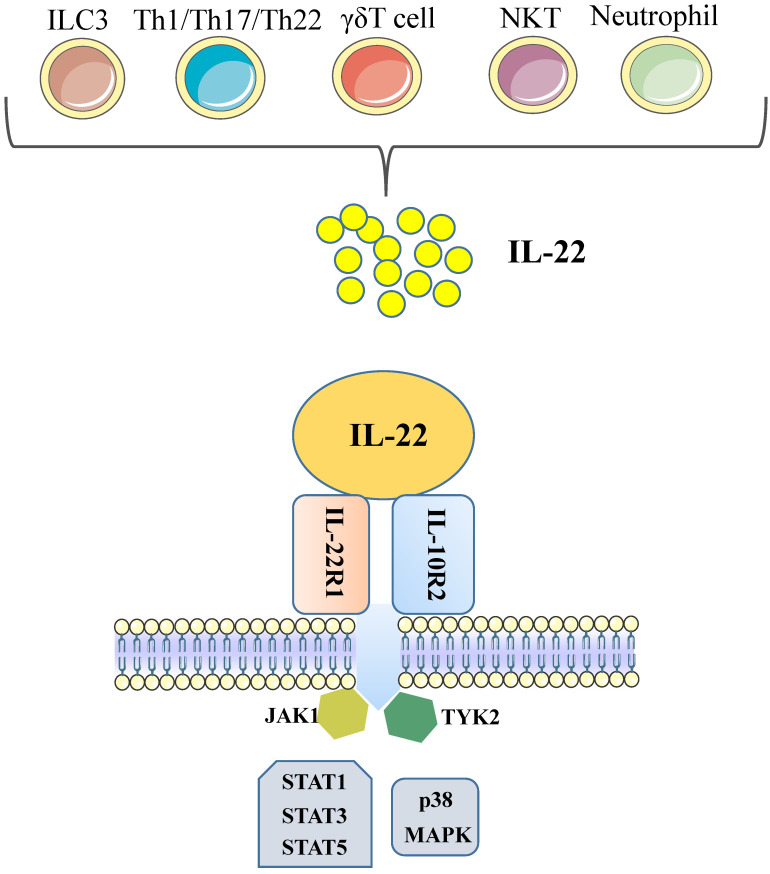
** Cellular sources and effects of IL-22.** IL-22 is produced mainly by cells of the hematopoietic system. During steady-state, ILC3 are the main producers of IL-22 in the mucosae of the large and the small intestine. During infections, Th1/Th17/Th22 cells will expand and produce amounts of IL-22. In addition, γδT cells and NKT cells have also been shown to capable of producing IL-22. Some reports have also found that neutrophils produce IL-22. IL-22 is a member of the IL-10 family of cytokines, all of which share common features in their receptors. Functional IL-22R is a heterodimer composed of IL-22R1 and IL-10R2. Each of these receptors signals through the Jak1/Tyk2-STAT pathway, although there is evidence that IL-22 can also signal through MAPK and p38 pathways. (ILC, innate lymphoid cell; Th, T helper cell; NKT, natural killer T cell; IL, interleukin; STAT, signal transducer and activator of transcription; MAPK, mitogen-activated protein kinases).

Although the production of IL-22 is limited to hematopoietic cells, mainly the lymphoid system, the IL-22 receptor( IL-22R) complex appears to be restricted to non-hematopoietic cells. Functional IL-22R is a heterodimer composed of IL-22R1 and IL-10R2, the latter is shared with various cytokine receptor complexes of the IL-10 family 12, 13. IL-10R2 is commonly expressed, while IL-22R1 is believed to only be expressed in the epidermal cells of various organs (such as bronchial, liver, pancreas and intestinal) and stroma cells 14, 15. In the liver, hepatocytes express both IL-10R2 and IL-22R1; Many diseases, such as psoriasis, graft-versus-host disease, liver and pancreas damage, ulcerative colitis and tumours are closely related to the IL-22 -IL-22R1 system, this point has been demonstrated in the experimental data in recent years 12. Upon binding of IL-22 to the IL-22 receptor-associated Jak1/Tyk2 kinases are activated resulting in phosphorylation of these receptors and activator of transcription (STAT)1, STAT3, STAT5 proteins. While STAT3 phosphorylation is the primary mediator of IL-22 signaling, STAT1 and STAT5 also show phosphorylation to some extent. STAT3 induces the expression of many genes involved in several signalling pathways, including pathways involved with apoptosis, the cell cycle and others. In addition to the STAT signaling, the IL-22-IL-22R1-IL-10R2 complex also results in activation of the mitogen-activated protein kinases (MAPK) and p38 pathways 12, 16-18 (Figure 1). One of the key regulators of IL-22 signaling is IL-22 binding protein (IL-22BP), which is a soluble form of the IL-22R1 subunit. Il-22BP binds IL-22 where it overlaps IL-22R1, thus interfering directly with the binding of the membrane-bound receptor. The affinity of IL22 to this soluble receptor (IL22BP) is 1, 000-fold higher than that of the membrane receptor (IL22R1). The effects of IL22 are inhibited directly by binding of IL22 to IL22BP. IL22BP was expressed in lung, pancreas, skin and other tissues as well as in DC, MQ and epithelial cells 7, 19, 52.

### IL-22 and liver injury

#### Alcoholic liver injury

Chronic alcohol consumption is a major cause of chronic liver disease, causing a wide range of disorders, from fatty liver disease to liver fibrosis and cirrhosis 20. Alcoholic liver disease (ALD) is a complicated process and causes a wide range of liver damage. The pathogenesis mainly includes direct hepatotoxicity caused by ethanol and its metabolites, oxidative stress caused by ethanol metabolism, elevation of pro-inflammatory cytokines and chemokines, activation of innate immune system, and other mechanisms. In the early stages of ALD, controlled diet and nutritional interventions have proven to be beneficial in curing most patients with mild ALD, but cannot cure severe forms of ALD. Corticosteroids, which were first utilised in the early 1970s for the treatment of severe alcoholic liver disease, are still the only drugs currently used for the treatment of this disease. However, hormone therapy may lead to many negative side effects, including the inhibition of liver regeneration and the promotion of bacterial infections 21, 22.

**Figure 2:**
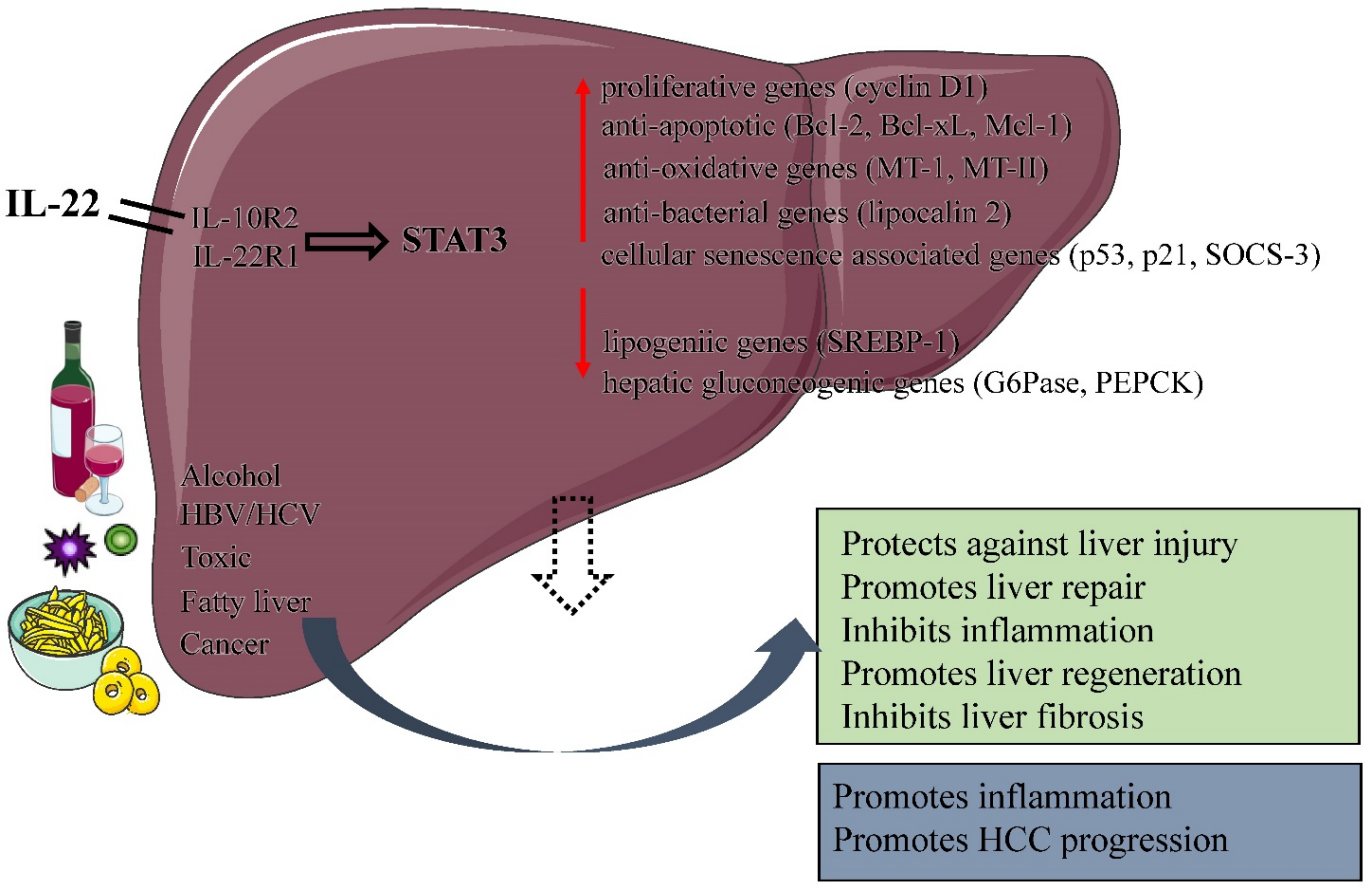
** Protective and pathological effects of IL-22.** IL-22 plays a number of protective roles in the liver by increasing the expression of anti-apoptotic, anti-oxidative, proliferative and anti-bacterial genes, including protecting against liver injury, Inhibiting liver fibrosis, promoting liver repair and regeneration. In addition, the function of IL-22 is dual nature: a pro-inflammatory activity in acute viral hepatitis infection; an anti-inflammatory activity in chronic viral hepatitis infection. IL-22 itself does not initiate HCC development but may promote HCC progression. (IL, interleukin; STAT 3, signal transducer and activator of transcription 3; HBV, Hepatitis B virus; HCV, Hepatitis C virus; SOCS-3, suppressor of cytokine signaling-3; HCC, Hepatocellular carcinoma ).

In the models of alcoholic liver injury (chronic-binge ethanol feeding, acute ethanol feeding), IL-22 has undoubtedly a protective effect on alcoholic fatty liver and related liver damage 6, 23, 24. IL-22 ameliorats steatosis and hepatocellular damage via the activation of STAT3 in hepatocytes during early alcoholic liver injury; and activation of STAT3 subsequently leads to the upregulation of anti-oxidative (related to MT-1 and MT-2 gene), anti-apoptotic (related to Bcl-2, Bcl-xL and Mcl-1 gene) and anti-bacterial genes (related to lipocalin 2 gene) (Figure 2). It also leads to the downregulation of transcription factors related to lipid biosynthesis.* In vivo* or* in vitro*, treatment of IL-22 can promote hepatocyte proliferation or liver regeneration, respectively 25. It has been clearly demonstrated that IL-22 was able to preventing the occurrence of bacterial infections and acute kidney injury (AKI), which are the main causes of death in patients with alcoholic hepatitis (AH) 22. In a mouse AKI model, IL-22 treatment was used to improve the survival and regeneration of renal tubular epithelial cells, which significantly improved the ischemia and reperfusion injury; On the other hand, IL-22 plays an important role in the host defence against invading pathogens by stimulating epithelial cells to produce antimicrobial proteins. Administration of IL-22 combine with steroid may be beneficial in the treatment of severe alcoholic hepatitis as IL-22 can negate the side effects of hormone therapy and contribute to the regeneration of the liver. A recent study from Dr. Schnabl's lab showed that ethanol-associated dysbiosis reduced intestinal IAA levels, activated AHR, and decreased intestinal IL-22 expression, leading to a decrease in REG3G expression. The result is that bacterial translocation to the liver and steatohepatitis. Bacteria engineered to produce IL-22 can induce REG3G expression to reduce ethanol-induced steatohepatitis 26.

#### Nonalcoholic fatty liver injury

Nonalcoholic fatty liver disease (NAFLD) ranges from simple steatosis to nonalcoholic steatohepatitis (NASH), cirrhosis, and even hepatocellular carcinoma. Prevalence of NAFLD in Asia is currently about 25%, similar to many western countries 27. Although progress has been made in the potential mechanisms of NAFLD and in the identification and development of new therapeutic targets, many problems remain to be resolved and there are no approved therapeutic drugs 28. IL-22 plays a particular role in alleviating the progression of NAFLD 29.

Blueberries combined with probiotics (BP) have anti-inflammatory and anti-apoptotic properties and may be potential candidates for NAFLD treatment. Using rat NAFLD model, Zhu J et al. 30 found that IL-22 participated in BP therapy by activating JAK1/STAT3 signaling pathway and inhibiting apoptotic factor BAX. In the presence of IL-22, BP could significantly reduce the accumulation of liver lipid droplets and triglyceride (TG), while in the absence of IL-22, lipid droplets and TG levels were significantly increased. Administration of IL-22 relieved metabolic syndrome in obese mice, resulting in reduction of weight and epididymal fat-pad mass, improvement of glucose and insulin tolerance, and regulation expression of lipogeniic genes 31. Additionally, IL-22 reduced the elevation of serum alanine aminotransferase (ALT) and aspartate aminotransferase (AST) levels induced by HFD, and partially inhibited the upregulation of lipid-related genes involved in liver lipid synthesis. Activation of JAK1/STAT3 signaling pathways might account for the protective action of IL-22 against fatty liver 32, 33.

#### Toxic liver injury

Many toxic substances cause different types of liver damage, both acute injury and chronic injury. Researchers typically use the chemical substances concanavalin A (ConA), Carbon tetrachloride (CCl4), lipopolysaccharide (LPS)/D-galactosamine(GaIN) to induce liver injury in mice and thereby simulate different types of human liver injuries 34-36. IL-22 has been shown to activate a variety of signal transduction pathways, including the activator of transcription factor (STAT) pathway, the Janus kinase-signal transducer pathway and the mitogen-activated protein kinase pathway in hepatic cells. IL-22 plays a protective role in T cell-mediated hepatitis by activating STAT3; Overexpression of IL-22 can significantly increase the activity of STAT3 and the induction of anti-apoptotic proteins such as Bcl-xL, Bcl-2 and Mcl-1 37 (Figure 2).

Just as we expected, as a survival factor for hepatocytes, IL-22 has a direct protective effect on acute liver injury induced by various chemical substances 38, 39. Hydrodynamic gene delivery of IL-22 prevented liver damage in ConA, CCl4 and Fas agonist liver injury models 4. Results have shown that the expression of IL-22 protein in the liver was significantly induced after injection of ConA. The blockade of IL-22 by neutralising antibodies can aggravate the liver damage induced by ConA and that treatment with recombinant IL-22 protein is sufficient to reduce the damage 37, 40. In a mouse model of liver injury induced by CCl4, the expression of IL-22 protein was also modestly increased 4, 37. The protective effect of IL-22 on liver injury induced by GaIN/LPS is also evident. Xing et al. revealed that IL-22 activates STAT3 after liver injury, increasing the expression of HO-1 and Ref-1, which both play a role in antioxidation. IL-22 treatment is also able to decrease the serum levels of ALT, total bilirubin (T.Bil) activity and improve liver histological markers, which reduces the mortality caused by GaIN/LPS 41. In acetaminophen (APAP)-induced liver injury (AILI) model, IL-22 pretreatment protected mice from APAP-mediated hepatotoxicity. Similarly, the protection was also dependent on STAT3. What's interesting is that short-term acute IL-22 exposure protects mice against AILI through STAT3 activation; however, chronic constitutive overexpression of IL-22 exacerbates AILI by increasing Cyp2E1 and toxic reactive APAP metabolite production 42. Mo R's lab revealed that enhanced AMP-activated kinase (AMPK)-dependent autophagy contributes to protective effects of IL-22 against APAP-induced liver injury. IL-22 administration distinctly upregulated hepatic LC3-II and p-AMPK; reduced serum ALT/AST, liver necrosis, and hepatic reactive oxygen species in APAP-challenged mice. When p-AMPK was blocked with AMPK inhibitor, IL-22-mediated LC3-II conversion and protection against APAP-induced cytotoxicity were decreased 43.

### IL-22 and inflammation

#### Hepatitis B

Hepatitis B virus (HBV) infection is a global public health threat, and is the major cause of hepatitis, liver cirrhosis, and hepatocellular carcinoma (HCC) 44. The outcome of HBV infection mainly depends on the balance between host and virus. The occurrence, development and pathogenesis of HBV infection is a complex process involving host innate and adaptive immunity, which often affects the efficacy of anti-HBV drugs 45. T-cell populations able to produce IL-22 are higher in patients with HBV hepatitis than in healthy subjects 46, 47, such as regulatory T, Thelper (Th)17, and Th22 cells. Similarly, the expression of IL-22 was significantly increased in patients with hepatitis B and the levels of IL-22 in serum or in the liver are closely related to the severity of liver disease in patients with Hepatitis B viral infection 48.

The role of IL-22 in HBV induced-liver inflammation remains controversial. It is possible that IL-22 has paradoxical dual nature of the anti-inflammatory or proinflammatory activity in HBV hepatitis. In acute HBV infection IL-22 may play a proinflammatory role by amplifying immune cell infiltration and clearance of the virus. In HBV transgenic mice model, injection of IL-22 increased the expression of pro-inflammatory genes in liver, and the transplantation of splenocytes from HBV-immunized mice into HBV transgenic mice significantly ameliorated the severity of liver injury in mice by neutralization of IL-22 47, 49. However, during chronic HBV infection, IL-22 may be mainly protective in some situations, yet pathogenic in others. In livers of mice and patients with chronic HBV infection, IL22 produced by inflammatory cells promoted proliferation of liver stem/progenitor cells (LPCs) via the activation of STAT3 48. Depletion of IL-22 significantly inhibited the expression of chemokines and recruitment of inflammatory cells into the liver, suggesting that IL-22 may be an important mediator by increasing the expression of chemokines to recruit inflammatory cells into the liver. Although IL-22 plays such an important role, IL-22 does not directly inhibit virus replication 47. In fact, chronic HBV infection is a complex process that includes immune tolerance (high HBV and low ALT), immune activity (high HBV and high ALT), and immune inactivity (low HBV and low ALT). IL-22 expression may correlate with high or low ALT levels, suggesting that the balance between IL-22's protective and pro-inflammatory effects may be tipped during chronic HBV infection 50.

#### Hepatitis C

Hepatitis C virus (HCV) threatens the quality of life of more than 350 million people worldwide. An effective immune response clears about 20% of HCV infections. However, without an adequate immune response, the virus will continue to replicate and cause a chronic infiltration of inflammatory cells into the liver 51. Similar to hepatitis B, IL22-producing Th17 cells also increased significantly in the liver of patients with chronic hepatitis C 52. The expression of hepatic IL-22 mRNA was higher in HCV hepatitis than that in cholestatic liver disease, although the serum IL-22 level was not significantly different between HCV hepatitis and the normal control group. Due to IL-22 has no significant effect on the expression of IFN-α/β and antiviral proteins MxA and 2', 5'-oligoadenylates synthesis, it has no relevant antiviral activity* in vitro* models of HCV replication and infection 53. Additionly, serum IL-22 levels were dramatically higher in the antiviral treatment group than in the untreated group, and its levels increased gradually with the improvement of treatment effect, suggests that IL-22 serum level can be used as a predictor of the efficacy of antiviral therapy in HCV patients 54. However, the role of IL-22 in the process of HCV fibrosis is still controversial. Sertorio M et al. demonstrated that high levels of IL-22 can alleviate liver fibrosis and portal hypertension in hepatitis C patients, while IL-22BP (IL-22 binding protein, the physiological inhibitor of IL-22) increases the risk to develop severe fibrosis 55. On the other hand, Wu LY et al. found that most IL-22 producing (+) cells in the liver were located in the liver fibrosis area of HCV patients with cirrhosis, and the increase in the number were positively correlated with the fibrosis staging score. IL-22 may participate in the fibrosis formation of HCV-related liver fibrosis by promoting the proliferation and activation of hepatic stellate cells (HSCs), inhibiting cell apoptos 56.

### IL-22 and liver fibrosis

Fibrosis is an internal reaction to chronic injury. Its function is to maintain the integrity of the organ despite widespread necrosis or apoptosis 57. Liver fibrosis is a result of chronic liver injury, and it is characterised by the activation of HSCs and an accumulation of extracellular matrix proteins 58. Long-term liver damage may cause fibrosis to develop into cirrhosis. Liver fibrosis can be caused by many diseases, such as chronic hepatitis B or C infection, autoimmune diseases, biliary tract disease, alcoholic fatty hepatitis as well as non-alcoholic fatty hepatitis 57. IL-22 plays a role in various liver diseases, including liver inflammation, liver fibrosis and cirrhosis 25, 49, 59. HSCs are the predominant type of cell that induces liver fibrosis after liver injury. HSCs express high levels of IL-10R2 and IL-22R1. Both* in vivo* and* in vitro* experiments demonstrate that IL-22 can inhibit the apoptosis of HSCs and promote their survival via the induction of the anti-apoptotic genes Bcl-2 and Bcl-xl. Surprisingly, IL-22 overexpression, either by IL-22 transgenic mice or exogenous administration of adenovirus expressing IL-22, is able to reduce hepatic fibrosis and accelerate the resolution of liver fibrosis during recovery 59. HSCs are activated after hepatic injury, then begin to express alpha smooth muscle actin (α-SMA) and produce a large quantity of collagen, eventually lead to fibrosis of the liver. The level of α-SMA expression decreased after treatment with IL-22, as well as increase the expression of proteins related to cellular senescence, including p53 at serine suppressor 15 (ser15 p-p53), p53, p21 and SOCS3 (suppressor of cytokine signalling 3). Administration of IL-22 increased the number of HSCs associated with SA-β-gal (senescence-associated-galactosidase). In addition, the signal transducer and activator of transcription 3 (STAT3) will be activated by IL-22, which promotes the senescence of HSCs by p53- and p21-dependent pathways, and knockdown of STAT3 will prevent IL-22 induced the senescence of HSCs. In other words, IL-22 can improve hepatic fibrosis by inducing the senescence of HSCs 59.

### IL-22 and liver regeneration

The liver is the only solid organ system capable of regeneration after tissue injury in mammals 60. Liver regeneration occurs both in the acute recovery of liver mass after resection and in the maintenance of liver mass after chronic injury, is an active area of research currently. Liver regeneration is a highly organised and orderly process of tissue growth caused by the loss of liver tissue; therefore, there is a large number of genes involved in the process of liver regeneration 61.

IL-22 is closely related to the proliferation of many types of cells, including epithelial cells, hepatic cells and other cell types 62, 63. The expression of IL-22 in the serum and IL-22Ra mRNA in hepatic of mice were significantly increased after 70% hepatectomy under general anaesthesia. Although administration of exogenous IL-22 did not increase the replication of hepatocytes before the partial hepatectomy, anti-IL-22 antibody treatment prior to surgery was able to significantly reduce hepatic cell replication 63. This suggests IL-22 may promote the regeneration of liver by increasing the replication of hepatocytes.

Treatment with IL-22 significantly increased the number of hepatocytes by inhibiting cell apoptosis and promoting mitosis to promote liver regeneration 64. Previous studies have shown that HepG2 cells stably transfected with IL-22 cDNA express high levels of cyclinD1 and c-myc; further* in vivo* and* in vitro* researches have demonstrated that the overexpression of cyclin D1 contributes to the replication and growth of hepatocytes, furthermore, c-myc plays an important role in IL-22 induced liver cell replication, suggesting that both cyclinD1 and c-myc may promote mitotic activity in hepatocytes. Just as we expected, the stimulating effects of IL-22 on liver regeneration are related to STAT3 activation 65. The levels of IL-6 in the liver may increases with the activation of STAT3 after a partial hepatectomy, suggestes that IL-6 and STAT3 are part of an early mechanism of liver cell proliferation.

There are two different forms of liver regeneration: hepatocyte regeneration and liver progenitor cells (LPCs) regeneration. LPC regeneration is largely responsible for liver regeneration after liver injury 66, 67. Progenitor cells have a high degree of regulation to regulate replication, as well as the ability to differentiate to achieve accurate tissue repair 61. In severe liver injury or chronic liver injury, if the mature liver cells cannot proliferate to repair the damage, LPC-mediated repair mechanisms will be used to maintain liver function. LPCs are able to differentiate into hepatocytes and bile duct epithelial cells 68-71, meanwhile express both IL-10R2 and IL-22R1. It has been proved that IL-22 has the ability to promote the proliferation of LPCs in a STAT3- dependent manner. Consequently, IL-22 can not only stimulate the proliferation of mature liver cells, but can also promote liver repair and regeneration in patients with severe or chronic liver injury by targeting LPCs 48. Hepatic IL-22 expression was increased in HBV patients and correlated with severity of liver inflammation and proliferation of LPCs. Proliferation of LPCs was increased significantly after 3, 5-diethoxycarbonyl-1, 4-dihydrocollidine (DDC) diet in overexpressed an IL-22 transgene specifically in liver (IL-22TG) mice. STAT3 was activated in LPCs isolated from DDC-fed IL-22TG mice. Proliferation of LPCs was decreased in liver with deletion of STAT3. IL-22R1 and IL-10R2 were also detected on LPCs isolated from DDC-fed wild-type mice. Treatment with IL-22 on LPCs activated STAT3 to induce these cells proliferation, but IL-22 had no effect on proliferation of LPCs with deletion of STAT3.

### IL-22 and Hepatocellular carcinoma

Hepatocellular carcinoma accounts for the majority of primary liver cancers. Worldwide, liver cancers are the fourth most common cause of cancer-related death and rank sixth in terms of incident cases 72. The 5-year survival rate for patients with liver cancer is only 18%, making it the second most deadly cancer after pancreatic cancer 73. Most HCC occurs in patients with underlying liver disease, mainly as a result of viral hepatitis or alcohol abuse. Currently, there are many treatments for HCC, such as surgical treatment, chemotherapy, radiotherapy, transcatheter chemoembolization/transcatheter chemoembolization (TACE), but for advanced patients, liver transplantation may be the only effective method. Unfortunately, due to the lack of donor, immune rejection, surgical injury and high cost, liver transplantation is not an ideal treatment 74, 75. The tumour and inflammatory microenvironment are considered to be the main battlefield between antitumor immunity and tumor promotion; it is very meaningful for the further understanding of the HCC with the new exploration of tumor microenvironment 76.

We know that IL-22 is a protective factor for the liver, by promoting the regeneration of tissue, increasing the function of the barrier, reducing the chronic inflammation and preventing the occurrence of cancer. In fact, IL-22 plays a dual role in the occurrence of HCC; On the one hand, it protects the liver from damage by contributing to the survival of normal hepatic cells; On the other hand, IL-22 can promotes the survival of damaged liver cells (which can be the precursor of hepatocellular carcinoma), ultimately leading to the occurrence of hepatocellular carcinoma 3. Compared with peripheral lymphocytes, IL-22 expression was significantly upregulated in human HCC tumor infiltrating leukocytes (TILs) 77. Moreover, IL-22 expression in Edmondson Grade III-IV HCC patients was observably higher than that in Grade I-II. IL-22 expression and STAT3 activation continued to increase in liver tissue in mice with chronic hepatitis and HCC models. Phosphorylation of STAT3 and up-regulation of downstream genes CyclinD1, Bcl-2, Bcl-XL, and vascular endothelial growth factor (VEGF) promote tumor growth and metastasis. In the diethylnitrosamine-induced HCC model, tumor formation was significantly reduced in IL-22 knockout mice. In conclusion, IL-22 plays a pro-tumor and anti-apoptosis role in HCC. More significantly, Oliver Waidmann 78 found that serum IL-22 levels is associated with disease severity in patients with advanced cirrhosis, and high serum IL-22 level is closely related to the short overall survival of patients with HCC verified by assessment of serum IL-22 level for 156 patients with HCC. IL-22 serum levels may reflect increased aggressiveness of liver cancer disease and act as a negative prognostic indicator in patients with HCC. It is important to note that although IL-22 is able to promote the proliferation and survival of liver cancer cells, IL-22 transgenic mice (with a high level of IL-22) cannot spontaneously induce hepatocellular carcinoma, suggesting that IL-22 itself does not lead to the development of HCC 5.

## Conclusions

Liver diseases remain a major global threat to human health 79. Cytokines are attractive therapeutic targets in liver diseases because of their importance in regulating immune and inflammatory responses 80-82. For instance, IL-6 have been shown to play an important role in alleviating steatosis and hepatocyte injury by activating STAT3 in hepatocytes during early alcoholic liver injury 83, 84. Although the hepatoprotective function of IL-6 has been fully demonstrated, the ubiquitous expression of IL-6 receptor makes many potential side effects of IL-6 in the treatment of liver diseases, which limits its clinical application 85. As an emerging CD4+Th cytokine, IL-22 plays an overwhelming hepatoprotective and regenerative roles in various liver diseases with the activation of STAT3 pathway 3. IL-22 has a protective effect on liver cells, can protects against liver injury, hepatitis, and liver fibrosis caused by a variety of reasons, and can promote liver regeneration after partial hepatectomy (Figure 2). What is worth mentioning is that IL-22 receptor expression is restricted to epithelial cells, which suggests that IL-22 treatment would likely produce fewer side effects than IL-6. IL-22 is expected to be a new attractive candidate for human liver diseases therapy.

A phase I trial of the recombinant human IL-22-Fc fusion protein (F-652) published by Tang et al. 86 shows minimal side effects in healthy volunteers. F-652 was well-tolerated in the first-in-man study following IV dosing. F-652 shown potent bioactivities, and did not induce pro-inflammatory cytokines/chemokines. Unlike the conventional anti-inflammation new drug, F-652 may be a first-in-class drug that plays an important role in protecting the survival and regeneration of tissues under immunological attack. A phase IIa trial is currently under way to determine whether IL-22-Fc treatment is also tolerated in patients with severe alcoholic hepatitis, and a phase IIb trial has been proposed for the treatment of severe alcoholic hepatitis 87. We look forward to the results of these clinical trials and hope that IL-22 therapy may benefit some patients with severe alcoholic hepatitis.
